# DeOri 10.0: An Updated Database of Experimentally Identified Eukaryotic Replication Origins

**DOI:** 10.1093/gpbjnl/qzae076

**Published:** 2024-10-15

**Authors:** Yu-Hao Zeng, Zhen-Ning Yin, Hao Luo, Feng Gao

**Affiliations:** Department of Physics, School of Science, Tianjin University, Tianjin 300072, China; Department of Physics, School of Science, Tianjin University, Tianjin 300072, China; Department of Physics, School of Science, Tianjin University, Tianjin 300072, China; Department of Physics, School of Science, Tianjin University, Tianjin 300072, China; Frontiers Science Center for Synthetic Biology and Key Laboratory of Systems Bioengineering (Ministry of Education), Tianjin University, Tianjin 300072, China; SynBio Research Platform, Collaborative Innovation Center of Chemical Science and Engineering (Tianjin), Tianjin 300072, China

**Keywords:** Replication origin, Eukaryote, DeOri, Database, DNA replication

## Abstract

DNA replication is a complex and crucial biological process in eukaryotes. To facilitate the study of eukaryotic replication events, we present a database of eukaryotic DNA replication origins (DeOri), which collects genome-wide data on eukaryotic DNA replication origins currently available. With the rapid development of high-throughput experimental technology in recent years, the number of datasets in the new release of DeOri 10.0 increased from 10 to 151 and the number of sequences increased from 16,145 to 9,742,396. Besides nucleotide sequences and browser extensible data (BED) files, corresponding annotation files, such as coding sequences (CDSs), mRNAs, and other biological elements within replication origins, are also provided. The experimental techniques used for each dataset, as well as related statistical data, are also presented on web page. Differences in experimental methods, cell lines, and sequencing technologies have resulted in distinct replication origins, making it challenging to differentiate between cell-specific and non-specific replication origins. Based on multiple replication origin datasets at the species level, we scored and screened replication origins in *Homo sapiens*, *Gallus gallus*, *Mus musculus*, *Drosophila melanogaster*, and *Caenorhabditis elegans*. The screened regions with high scores were considered as species-conservative origins, which are integrated and presented as reference replication origins (rORIs). Additionally, we analyzed the distribution of relevant genomic elements associated with replication origins at the genome level, such as CpG island (CGI), transcription start site (TSS), and G-quadruplex (G4). These analysis results can be browsed and downloaded as needed at http://tubic.tju.edu.cn/deori/.

## Introduction

In eukaryotes, errors arising from the DNA replication process can easily lead to a series of problems, including cell damage and cancerous changes. Therefore, the mechanisms underlying DNA replication initiation and regulation need to be better understood to explore cellular changes at the molecular level. In the three domains of life, all DNA replication is initiated from replication origins [1]. However, the diversity and complexity of eukaryotic replication origins make it challenging for researchers to study them in depth and comprehensively [2,3]. Based on numerous studies on *Saccharomyces cerevisiae* and *Xenopus laevis*, a theoretical system of eukaryotic DNA replication initiation mechanisms and regulatory systems has been preliminarily established. Although the origin of replication varies widely between phyla and species, the pre-RC mechanism is conserved in eukaryotes [4–6]. Thus, several experimental methods for replication origins have been developed, including sequencing by short nascent strands (SNS-seq), sequencing by Okazaki fragments (OK-seq), sequencing by chromatin immunoprecipitation (ChIP-seq), mapping the sequences of nascent DNA replication strands throughout the six cell cycle phases (Repli-seq), and sequencing by bubbles with restriction fragments containing replication initiation sites (Bubble-seq) [7–11]. However, the characteristics and definitions of replication origins differ slightly among species. For example, the origin of replication in budding yeast is a sequence-specific autonomously replicating sequence (ARS) consensus sequence (ACS) sequence with a length of 100–200 bp, whereas the origin of replication in metazoans is not sequence-specific, and its length can vary from 200 bp to a 1-Mb replication domain [12,13]. Although scientists have greatly promoted statistical analysis and data mining of replication origins, existing databases have not yet integrated massive amounts of data. Moreover, biases in samples and experimental methods, such as different protein conjugates, chromatin states, and different cell stages, have resulted in different replication origins obtained by sequencing. This complexity makes it difficult for researchers to integrate and extract the required data from numerous experimental results within a short timeframe.

To address these problems, DeOri database was developed a dozen years ago [14]. The initial version of DeOri provided 10 datasets and 16,145 sequences of replication origin. Henceforth, DeOri is constantly maintained and expanded. DeOri database has assisted experimental researchers in exploring eukaryotic replication mechanisms since its release [15], and many statistical and machine learning studies have used data from DeOri for related analysis and prediction [16–20]. Besides, DeOri has been considered as a landmark event in the study of eukaryotic replication origin [21]. Compared to the initial version, more data types, functions, and download files have been provided in the new version of DeOri 10.0 (**Table 1**). We not only increased the number of datasets to 151 and sequences to 9,742,396, but also counted the distributions of the transcription start sites (TSSs), CpG islands (CGIs), and G-quadruplex (G4) structures in the upstream and downstream 20 kb of sequences. The genomic features within the sequence regions in each dataset were also extracted. We then provided users with distribution curves, heatmaps, statistical information, and genome annotations of genomic elements in the sequence regions [*e.g.*, coding sequence (CDS), exon, mRNA, and TSS]. The user-friendly search modules BLAST and genome browser JBrowse were added to DeOri 10.0, allowing users to browse, select, and compare multiple datasets more conveniently and comprehensively [22,23]. Finally, we determined reference replication origins (rORIs) for five species with relatively conservative origins in large datasets.

**Table 1:** Comparison between DeOri 1.0 and DeOri 10.0

Feature	Details	DeOri 1.0	DeOri 10.0
Data type	Origin	**√**	**√**
	Zone		**√**
	Site		**√**
Function	Browse	**√**	**√**
	Search		**√**
	BLAST		**√**
	JBrowse		**√**
	Statistical analyses		**√**
Download file	BED		**√**
	FASTA	**√**	**√**
	Basic information	**√**	**√**
	mRNA annotation		**√**
	CDS annotation		**√**
	Exon annotation		**√**
	TSS annotation		**√**
	CGI annotation		**√**

*Note*: Type “Origin” refers to the replication origin with general definitions; type “Zone” refers to the region enriched with replication origins or a long replication initiation sequence obtained by OK-seq; type “Site” refers to the region that binds to specific replication-associated proteins (*e.g.*, CDC6, MCM2-7, and ORC1-2). CDS, coding sequence; CGI, CpG island; TSS, transcription start site.

## Database content and usage

### Data source and database content

As of December 19, 2023, DeOri had collected approximately 9.7 million sequences (*e.g.*, replication origins, replication zones, and protein-binding sites), which included 15 species, 60 cell types, and 151 datasets (**Table 2**). DeOri data were obtained from various experimental studies and publicly available databases. Most browser extensible data (BED) files and sequence data were obtained from Gene Expression Omnibus (GEO) [24], and other data were derived from the databases such as Trypanosomatidae Database (TriTrypDB) [25] and The Encyclopedia of DNA Elements Consortium (ENCODE) [26]. Reference sequence (RefSeq) and annotations were downloaded from University of California Santa Cruz (UCSC) [27], National Center for Biotechnology Information (NCBI) [28], WormBase [29], Database of *Drosophila* Genes & Genomes (Flybase) [30], and The *Arabidopsis* Information Resource (TAIR) [31]. Based on the RefSeq and annotations, DeOri 10.0 extracted the genome features for each dataset to provide annotation files that contain the features within each sequence. The annotations included CDS, exon, mRNA, TSS, and CGI, which are available on the download page as text files. DeOri also displays basic statistical information about the dataset, BED files, and FASTA files. For the data used for the analysis, TSS data were extracted from Reference Flat File Format (refFlat) files containing transcriptions in UCSC or General Feature Format (GFF) files containing reference annotations in NCBI; CGI data were predicted by the UCSC software cpg_lh (http://hgdownload.soe.ucsc.edu/admin/exe/linux.x86_64/cpg_lh), and G4 data were obtained from two databases: G4Bank [32] and EndoQuad [33]. Due to the differences in sequencing methods and experimental techniques in different literature, we followed several common bioinformatics software tools with parameters used in the original literature to process the raw data. The data processing flow is shown in Figure S1, which consists of four main procedures: (1) quality control; (2) RefSeq alignment; (3) processing Bam/Sam files; (4) peak calling. Additionally, we extracted FASTA files and genomic element annotations based on the BED files obtained from peak calling. The GC content, sequence length, and non-standard base content of sequences in the FASTA files were also calculated for users.

**Table 2:** Contents of DeOri 10.0

Organism	No. of cell types	No. of datasets (Origin)	No. of sequences (Origin)	No. of datasets (Zone^#^ or Site*)	No. of sequences (Zone^#^ or Site*)
*Arabidopsis thaliana*	3	5	12,744	1*	22,472*
Yeast	5	5	1448	0	0
*Homo sapiens*	30	54	4,074,391	31 (14^#^, 17*)	3,721,666 (108,040^**#**^, 3,613,626*)
*Mus musculus*	10	20	1,666,211	4^#^	43,761^**#**^
*Drosophila melanogaster*	4	4	22,294	1*	5135*
Nematode	7	22	67,359	1*	345*
*Gallus gallus*	1	2	101,557	1^#^	3013*

*Note*: Yeast in Organism contains five species including *Pichia pastoris*, *Saccharomyces cerevisiae*, *Candida glabrata*, *Schizosaccharomyces pombe*, and *Kluyveromyces lactis*; Nematode in Organism contains five species including *Caenorhabditis elegans*, *Leishmania major*, *Leishmania mexicana*, *Trypanosoma brucei*, and *Trypanosoma cruzi*. The numbers with ^#^ correspond to type Zone, and those with * correspond to type Site. Please refer to the note of Table 1 or the main text for the description of types Origin, Zone, and Site.

### Reference replication origins

**Figure 1:**
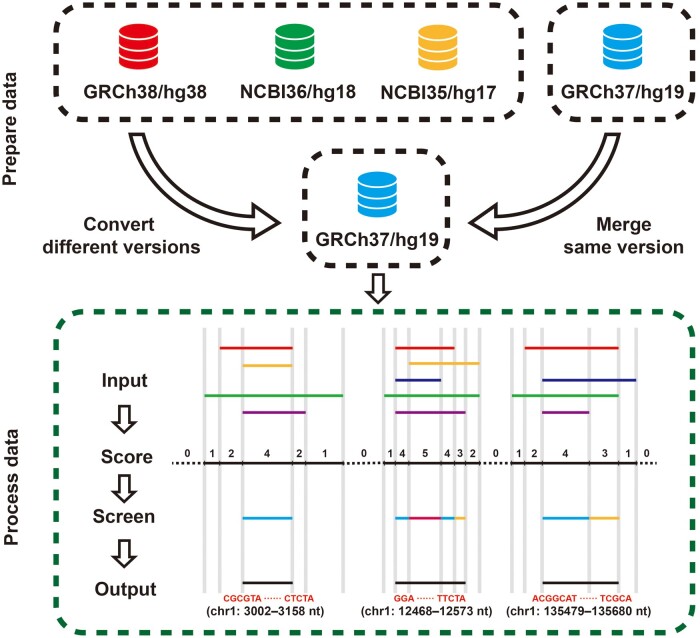
Extraction process of rORIs Prepare data: converting datasets of multiple different genome versions in an organism into the same version. Process data: (1) input multiple files of the same genome version after conversion, and use multiinter of BEDTools to score the overlapping parts of multiple datasets; (2) get the scored file; (3) screen the regions that are no less than 1/2 of the maximum overlapping number in the chromosome (rounded up) as the conservative replication origin; (4) merge the adjacent fragments and output the BED file and sequences.

Despite the extensive data collection in DeOri, users still face challenges in filtering and utilizing such a vast and diverse dataset. Therefore, DeOri provides users with five relatively conservative rORIs for eukaryotic datasets. During data preparation for rORIs, multiple different genome versions were transformed into the same genome version using liftOver (https://genome.ucsc.edu/cgi-bin/hgLiftOver). Subsequently, the overlapping regions were scored using multiinter in BEDTools [34]. For each chromosome, only regions with scores no less than half of the maximum overlap number (rounded up) were added to the reference dataset (**Figure 1**). Currently, reference datasets for *Homo sapiens, Gallus gallus, Mus musculus, Drosophila melanogaster*, and *Caenorhabditis elegans* are available in DeOri. Based on these criteria, rORIs are regarded conservative and non-species-specific. Therefore, the rORIs are recommended for consideration when researchers pay more attention to sequence conservation of replication origins. In addition, rORIs can be treated as non-specific, and the origins beyond rORIs are potentially cell-specific.

### Statistics on replication-related genomic elements

The statistical analysis on the distributions of genomic elements, such as TSS, CGI, G4, and gene, was conducted at a broader scale (within the range of –20 kb to 20 kb from center of replication-related sequences) and a longer step size (1 kb), which would provide researchers with a landscape for further investigation. In data preparation, the files containing the location information of genomic elements are converted into bigwig format. In data calculation, the distribution matrix between each dataset and genomic elements is calculated by the computeMatrix of deepTools [35], and then the NaN value in the matrix is changed to zero. Subsequently, the columns of each matrix are averaged, and the obtained results are normalized with the scale of 0–5. Finally, the heatmap is drawn based on the calculation results mentioned above. In the statistical analysis of human replication origins, datasets are merged and compared using merge and intersect of BEDTools, and the correlation coefficient is calculated using jaccard of BEDTools.

## Database implementation and recent updates

### Database implementation

DeOri internal framework was built using MySQL (v8.0.35, http://www.mysql.com/) and Django (v4.2.5, https://www.djangoproject.com/). Jinja2 (v3.1.2), Bootstrap (v5.3.0, https://getbootstrap.com/), EChart (v5.4.3, https://echarts.apache.org/), jQuery (v3.7.1, https://jquery.com/), and DataTables (v1.13.6, https://datatables.net/) were used for page optimization and data visualization. The website was deployed and served by Nginx (v1.20.0) and Gunicorn (v21.2.0). Part of the picture material comes from Smart Servier Medical Art (https://smart.servier.com/).

### Updated contents and functions

Compared with the initial version, DeOri 10.0 has greatly improved and changed in data and interface (Table 1). DeOri 10.0 has been redesigned as a user-friendly and intuitive web page using the Django framework (Figure S2). There are three types of standardized data in the latest release of DeOri: Origin, Zone, and Site. The data format is slightly different, and these data type descriptions follow the original styles in the literature. “Origin” refers to the replication origin with general definitions, whose longest sequence is about 30 kb and the average length is 0.2 kb to 5 kb in different datasets; “Zone” refers to the region enriched with replication origins or a long replication initiation sequence obtained by OK-seq, whose longest sequence is about 170 kb and the average length is 20 kb to 30 kb; “Site” refers to the region that binds to specific replication-associated proteins (*e.g.*, CDC6, MCM2-7, ORC1-2). Besides the original data, DeOri also provides datasets of rORIs in five species for users, which are believed to be relatively conservative. Taking human rORIs as an example, to verify their conservation, we extracted and screened human replication origins from datasets with no less than 10,000 sequences and compared them with human rORIs. If the rORI overlaps with the replication origins in certain dataset, this rORI is considered as overlapping rORI. The average overlap ratio is about 60%. Consequently, the percentage of overlapping rORIs to the total rORIs could indicate the conservation of rORIs and the specificity of certain datasets to some extent (Table S1). For example, although both GR00030057 and GR00030058 are immortalized HMEC cell lines, differences in the mis-expression of genes cause significant differences in their shared percentages of rORIs.

Visualization of the dataset and sequences was presented in JBrowse. To improve the intuitiveness and convenience of DeOri, a simple home page and several modules for batch download, search, JBrowse, and BLAST are provided (Figure S3). Among these modules, JBrowse is a powerful genome browser that allows users to compare and contrast multiple datasets. It is embedded in the dataset page and the sequence page that demonstrates data in the JBrowse window. It can also be displayed for data browsing and comparison on a separate page. Furthermore, one concrete example is presented on the JBrowse page.

## Data statistics

### Statistical analysis results based on DeOri 10.0

**Figure 2:**
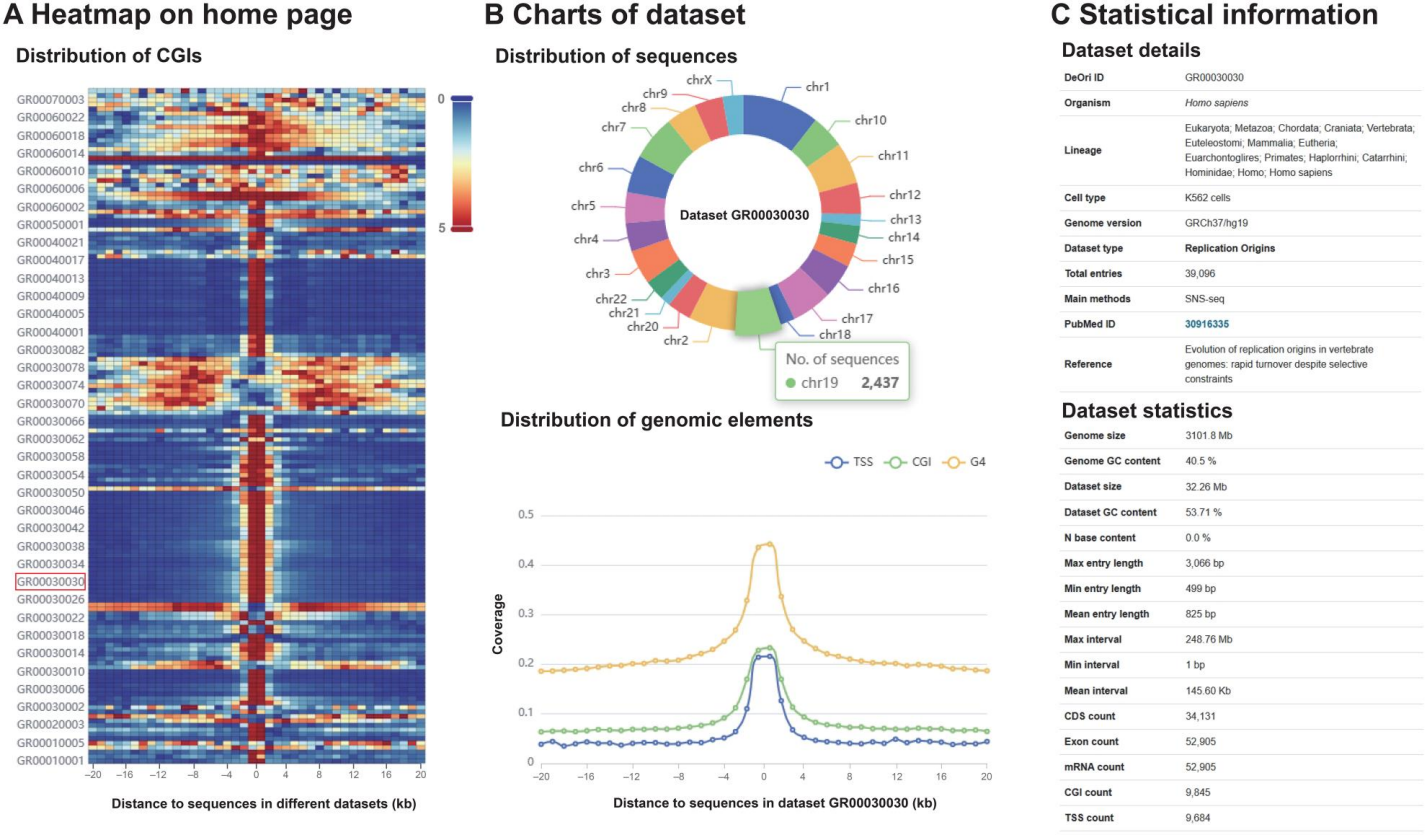
General information on datasets in DeOri **A**. Heatmap on the home page. Comparison of CGI distribution among different datasets. **B**. Charts of dataset. Visualization of statistics of GR00030030 dataset, such as the distributions of sequences and genomic elements. **C**. Statistical information. Basic information and statistical results on GR00030030 dataset. CGI, CpG island; TSS, transcription start site; G4, G-quadruplex.

The replication origins of eukaryotes are related to particular biological elements such as CGI, TSS, and G4 structure [36,37]. Therefore, the relevant statistical analyses were performed using DeOri 10.0. The statistical results of the relationships between these elements and the origins are presented on different pages. Users can gain an overview through the heatmap on the home page (**Figure 2**A) and view the data and features more intuitively on the dataset page (Figure 2B and C). Besides, these statistical results on dataset page can be used as one of the reference factors for selecting data. Take the record GR00030030 as an example [38], which is a replication-origin dataset of the human K562 cell line. The genomic elements of TSS, CGI, and G4 are highly concentrated within the 2-kb upstream and downstream regions from the center of replication origins in dataset GR00030030 (Figure 2B). These statistical results not only assist users in obtaining the differences between multiple datasets, but also help conduct further analysis.

### Characteristic analysis in human replication origins

**Figure 3:**
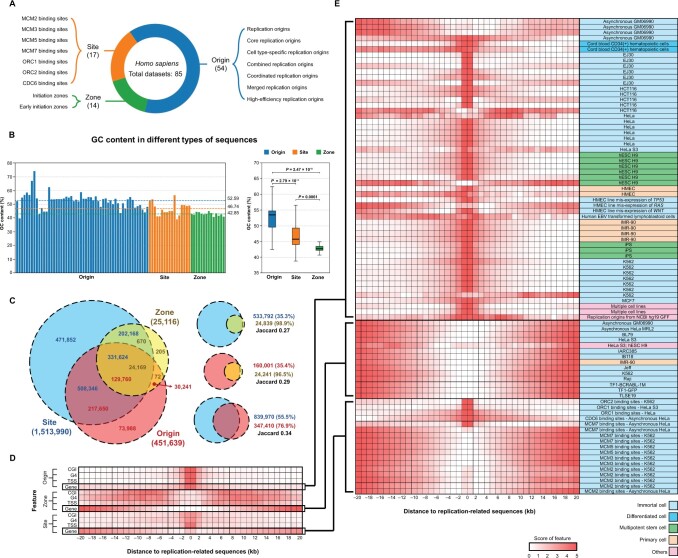
Characteristic analysis of human replication-related sequences **A**. Statistic of human replication-related datasets in DeOri 10.0. **B**. GC content in three types of sequences. **C**. The Venn diagram of the three types of sequences. **D**. Distributions of four genomic elements around the replication-related sequences. **E**. Statistics on the distributions of genes around the replication-related sequences in each dataset.

To demonstrate how DeOri can be used to enhance the understanding of eukaryotic replication origins, we conducted analysis of replication-related sequences in 85 human datasets, which have been categorized into three types: Origin, Zone, and Site in DeOri 10.0 (**Figure 3**A). It reveals that the GC content of type Origin is higher than that of types Site and Zone, with the latter exhibiting the lowest GC content (Figure 3B). In order to clarify the relationship among different types, the sequences of same type were merged together, which resulted in 451,639 sequences of type Origin, 1,513,990 sequences of type Site, and 25,116 sequences of type Zone. The number of shared sequences among the three types was counted, and the Jaccard correlation coefficients were calculated, respectively (Figure 3C). To explore the relationship between the replication-related sequences and other genomic elements, the distributions of the genomic elements, such as CGI, G4, TSS, and gene, were calculated within the range of –20 kb to 20 kb from the center of these sequences, respectively (Figure 3D). Consequently, there were highly concentrated distributions of CGI, G4, TSS, and gene within the range of –2 kb to 2 kb for type Origin, while there were discrete distributions at both ends for type Zone. As to type Site, there were highly concentrated distributions of CGI, G4, and TSS within the range of –2 kb to 2 kb, while there were no concentrated distribution of gene. Then, distributions on gene within each dataset were further calculated (Figure 3E). Consequently, there were still highly concentrated distributions of gene in different cell lines for ORC1, ORC2, and CDC6 binding sites, while there was only scattered distribution at both ends for MCM2-7 binding sites (Figure 3E). Furthermore, for all datasets, the cell lines were classified into five categories: immortal cells, differentiated cells, primary cells, multipotential stem cells, and others. It is evident that regardless of the cell lines, the majority of datasets demonstrate gene concentration within the range of –2 kb to 2kb from center of replication origins.

## Conclusion and perspective

Identifying the process underlying replication origins and regulatory mechanisms has been one of a focal point in replication origin research. The proposal and validation of pre-RC and CMG mechanisms have been reported [39]. However, several questions remain unanswered. On the one hand, with the explosive growth of origin-related datasets in eukaryotes, it has been a great impetus for researchers to explore the origin characterization, firing and regulatory mechanisms of replication origins through computational analysis and prediction. However, there have been only a few database studies on eukaryotic origins, such as OriDB [40], which summarizes the replication origins of yeast. To fill this gap, we constructed and developed DeOri, which not only provided a large amount of experimental data, but also presented the rORIs and statistical results. In practical application, researchers can compare the replication origins of different species or different cell lines based on data of DeOri, and explore their differences in sequence characteristics, chromatin distribution, *etc*. For computational science, sequence features can be extracted by machine learning to predict and distinguish the replication origins. For experimental science, the experimental methods and data processing shown in DeOri can be used as a reference. Additionally, based on the present analysis, there is highly concentrated distribution of genes around replication origins in human, which suggests a new analytical perspective for the researchers. Subsequent analysis could focus on the specific types and functions of genes enriched in replication origins. A deeper understanding of the human replication origins may reveal differences between normal and cancer cells, potentially leading to novel treatment methods and directions.

With the rapid development of artificial intelligence in recent years, the scientific research paradigm has been significantly promoted and changed [41]. In many studies on replication origins, machine learning has been used based on the available experimental data [42–45]. For example, replication origins in *Saccharomyces cerevisiae* genomes can be predicted using the support vector machine (SVM) method [46], and deep learning can be employed to identify replication origins in humans [47]. Large amounts of high-quality data are urgently required for high-quality computational science studies. We believe that DeOri will contribute to computational data mining and further understanding of the DNA replication process in eukaryotes. Furthermore, DeOri is expected to play a crucial role in experimental and computational studies of eukaryotic replication origins. In the future, we will continue to maintain and update DeOri timely, and further integrate latest experimental data to facilitate a comprehensive study of eukaryotic replication origins. With the development of experimental technology, we are expected to add more replication-related data, such as replication timing and epigenetic modification sites, and provide online service for data analysis in the next update.

## Supplementary Material

qzae076_Supplementary_Data

## Data Availability

DeOri is an open access database freely available at http://tubic.tju.edu.cn/deori/.

## CRediT author statement

**Yu-Hao Zeng**: Data collection, Statistical analysis, Writing – original draft, Visualization. **Zhen-Ning Yin**: Software, Writing – original draft, Visualization. **Hao Luo**: Data review, Writing – original draft, Visualization. **Feng Gao**: Conceptualization, Writing – review & editing, Supervision, Funding acquisition. All the authors have read and approved the final manuscript.
