# Supplementary material for: DeOri 10.0: An Updated Database of Experimentally Identified Eukaryotic Replication Origins
Source: Genomics Proteomics Bioinformatics. 2024 Oct 15;22(5):qzae076. doi: 10.1093/gpbjnl/qzae076 (PMC11652270; doi:10.1093/gpbjnl/qzae076)
Supplement: qzae076_Supplementary_Data [file qzae076_supplementary_data.zip › supplementary material captions.docx]

**Supplementary material**

**Figure S1 Data processing flow with related software and files**

**Figure S2 Data content of DeOri 10.0**

**A.** Browse organism. Presentation of the information and statistical results on species included in DeOri. **B.** Organism details. Demonstration of the information of datasets contained in the species (*e.g.*, cell lines, experimental methods, literature sources). **C.** Dataset page. Demonstration of the distribution of datasets, statistical information, and genome files. **D.** Sequence page. Display of details of certain sequence and the dataset on web page.

**Figure S3 Function pages in DeOri 10.0**

**A.** Home page. A basic overview of DeOri. **B.** Download page. Presentation of datasets for data download. **C.** Search page. Field search and BLAST based on the DeOri database. **D.** JBrowse2. An example and the entrance of JBrowse.

**Table S1 Comparison of overlap between different datasets and rORIs in human**
