# Supplementary material for: DeOri 10.0: An Updated Database of Experimentally Identified Eukaryotic Replication Origins
Source: Genomics Proteomics Bioinformatics. 2024 Oct 15;22(5):qzae076. doi: 10.1093/gpbjnl/qzae076 (PMC11652270; doi:10.1093/gpbjnl/qzae076)
Supplement: qzae076_Supplementary_Data [file qzae076_supplementary_data.zip › Table S1.docx]

**Table S1 Comparison of overlap between different datasets and rORIs in human**

| **DeOri ID** | **No. of dataset sequences** | **No. of rORIs** | **No. of overlapping rORIs** | **Overlap ratio* (%)** |
| --- | --- | --- | --- | --- |
| GR00030003 | 62,971 | 34,996 | 9,970 | 28.49 |
| GR00030004 | 94,195 | 34,996 | 13,434 | 38.39 |
| GR00030005 | 52,251 | 34,996 | 10,443 | 29.84 |
| GR00030006 | 100,301 | 34,996 | 22,631 | 64.67 |
| GR00030007 | 119,577 | 34,996 | 25,248 | 72.15 |
| GR00030008 | 23,820 | 34,996 | 15,866 | 45.34 |
| GR00030009 | 10,160 | 34,996 | 7,767 | 22.19 |
| GR00030010 | 25,054 | 34,996 | 11,454 | 32.73 |
| GR00030013 | 123,274 | 34,996 | 24,047 | 68.71 |
| GR00030014 | 34,172 | 34,996 | 17,187 | 49.11 |
| GR00030015 | 39,962 | 34,996 | 14,570 | 41.63 |
| GR00030016 | 36,219 | 34,996 | 13,923 | 39.78 |
| GR00030027 | 41,698 | 34,996 | 22,565 | 64.48 |
| GR00030028 | 39,300 | 34,996 | 20,244 | 57.85 |
| GR00030029 | 39,755 | 34,996 | 20,443 | 58.42 |
| GR00030030 | 39,096 | 34,996 | 20,308 | 58.03 |
| GR00030031 | 39,386 | 34,996 | 20,449 | 58.43 |
| GR00030032 | 39,248 | 34,996 | 20,405 | 58.31 |
| GR00030033 | 115,935 | 34,996 | 30,614 | 87.48 |
| GR00030034 | 67,103 | 34,996 | 29,068 | 83.06 |
| GR00030035 | 84,020 | 34,996 | 30,073 | 85.93 |
| GR00030036 | 39,427 | 34,996 | 20,604 | 58.88 |
| GR00030037 | 166,496 | 34,996 | 31,289 | 89.41 |
| GR00030038 | 87,690 | 34,996 | 23,800 | 68.01 |
| GR00030039 | 143,440 | 34,996 | 27,860 | 79.61 |
| GR00030040 | 68,204 | 34,996 | 27,351 | 78.15 |
| GR00030041 | 151,396 | 34,996 | 30,991 | 88.56 |
| GR00030042 | 152,159 | 34,996 | 30,978 | 88.52 |
| GR00030043 | 85,870 | 34,996 | 30,138 | 86.12 |
| GR00030044 | 74,783 | 34,996 | 29,192 | 83.42 |
| GR00030045 | 128,090 | 34,996 | 31,619 | 90.35 |
| GR00030046 | 70,766 | 34,996 | 27,480 | 78.52 |
| GR00030047 | 155,395 | 34,996 | 31,744 | 90.71 |
| GR00030048 | 316,983 | 34,996 | 29,776 | 85.08 |
| GR00030049 | 63,164 | 34,996 | 22,207 | 63.46 |
| GR00030050 | 73,905 | 34,996 | 20,605 | 58.88 |
| GR00030052 | 95,928 | 34,996 | 21,635 | 61.82 |
| GR00030054 | 37,357 | 34,996 | 11,620 | 33.20 |
| GR00030056 | 90,092 | 34,996 | 5,202 | 14.86 |
| GR00030057 | 107,637 | 34,996 | 22,180 | 63.38 |
| GR00030058 | 110,624 | 34,996 | 9,690 | 27.69 |
| GR00030059 | 147,241 | 34,996 | 25,672 | 73.36 |
| GR00030060 | 184,358 | 34,996 | 32,259 | 92.18 |
| GR00030061 | 40,720 | 34,996 | 14,031 | 40.09 |
| GR00030065 | 124,492 | 34,996 | 27,121 | 77.50 |
| GR00030066 | 44,482 | 34,996 | 19,931 | 56.95 |
| GR00030067 | 78,820 | 34,996 | 17,907 | 51.17 |
| GR00030068 | 36,903 | 34,996 | 3,822 | 10.92 |

*Note*: * Overlap rate is calculated by dividing No. of overlapping rORIs by No. of rORIs.
