# Supplementary figures and images for: DeOri 10.0: An Updated Database of Experimentally Identified Eukaryotic Replication Origins
Source: Genomics Proteomics Bioinformatics. 2024 Oct 15;22(5):qzae076. doi: 10.1093/gpbjnl/qzae076 (PMC11652270; doi:10.1093/gpbjnl/qzae076)

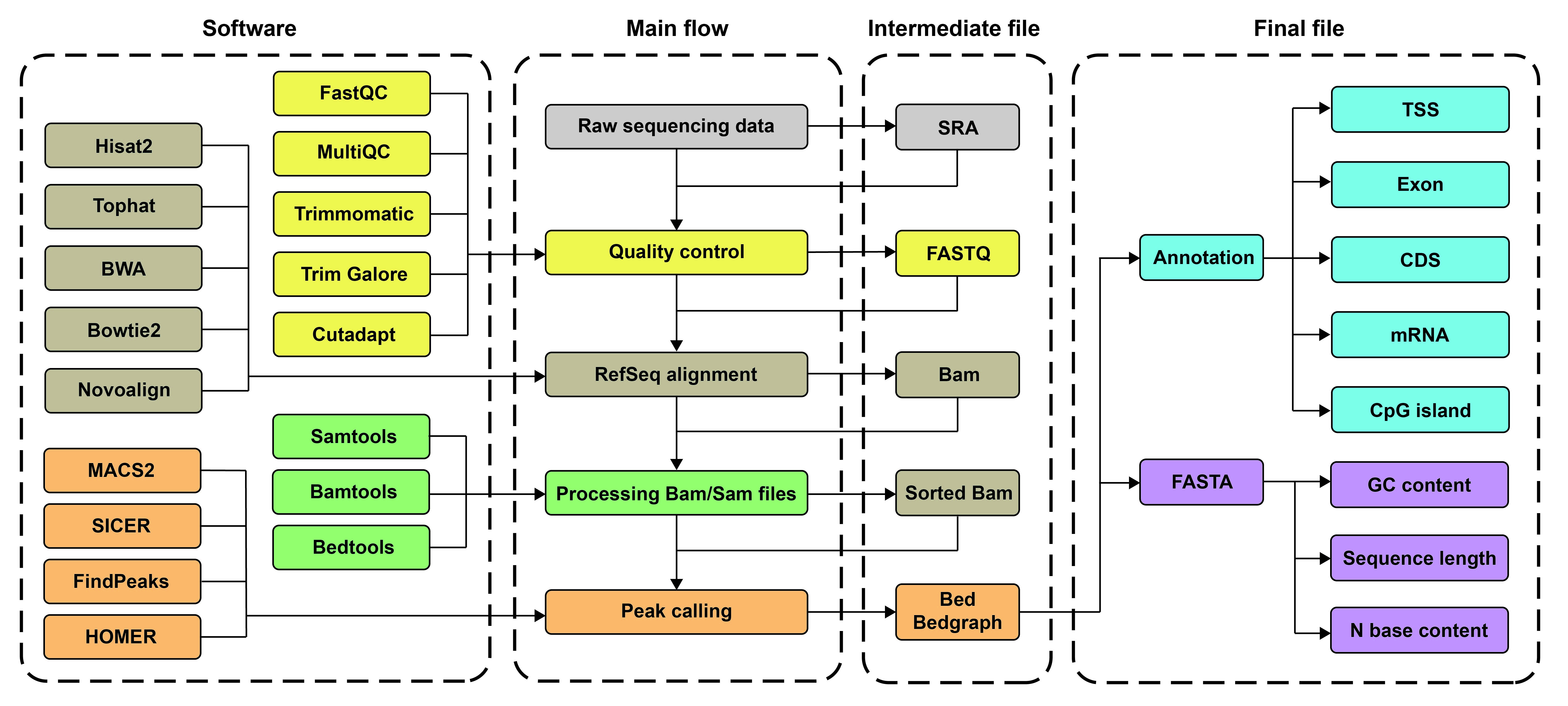

Supplement: qzae076_Supplementary_Data [file qzae076_supplementary_data.zip › Figure S1.jpg]
